# Peritoneal loose body: a possible cause of bowel perforation during PD catheter insertion

**DOI:** 10.1080/0886022X.2022.2075278

**Published:** 2022-05-23

**Authors:** Ning Yang, Shenglin Zhang, Ming Fang, Keping Wang, Hongli Lin, Longkai Li

**Affiliations:** a Graduate School of Dalian Medical University, Dalian, People’s Republic of China; b Department of Nephrology, Liaoning Translational Medicine Center of Nephrology, First Affiliated Hospital of Dalian Medical University, Dalian, People’s Republic of China; c Department of General Surgery, First Affiliated Hospital of Dalian Medical University, Dalian, People’s Republic of China

Dear Editor,

Perforation of bowel is a rare but recognized complication of peritoneal dialysis (PD) catheter placement [1] that may be difficult to avoid even for the experienced doctor, especially when using percutaneous needle-guidewire PD catheter insertion. Its incidence varied from 0.7% to 2.6% [1] although catheter insertion techniques have improved nowadays. In addition to operation procedures, there are also other causes of bowel perforation such as diseases of the bowel, abdominal wall adhesions [2], and other unknown reasons.

From a physical perspective, the bowel would be easily injured during percutaneous needle-guidewire catheter insertion if there is a solid fulcrum beneath the bowel, such as a peritoneal loose body (PLB). The PLB is generally a solid mass with smooth surface and gray or white color, the origin of which is thought to be torsed, infarcted, and detached epiploic appendages that slowly turn into fibrotic masses, which can move freely in the cavity [3]. The PLB is rare and – as far as we know – not reported previously in PD patients. Here, we present a case with bowel perforation which we believe was caused by a peritoneal loose body, which served as a supporting point under the sigmoid during percutaneous PD catheter insertion.

## Case presentation

A 77-year-old man was admitted to the hospital because of congestive heart failure due to fluid overload from chronic renal failure. After the patient achieved a stable clinical condition, PD was chosen, and a straight Tenckhoff catheter was inserted softly and slowly by an experienced nephrologist into the peritoneal cavity percutaneously by the Seldinger technique. Catheter flow tests performed during and after the catheterization showed inflow 1000 mL and outflow 950 mL, indicating that the PD catheter functioned well without flow obstruction. The effluent was clean and the patient did not report any complaints at that time.

**Figure 1.**
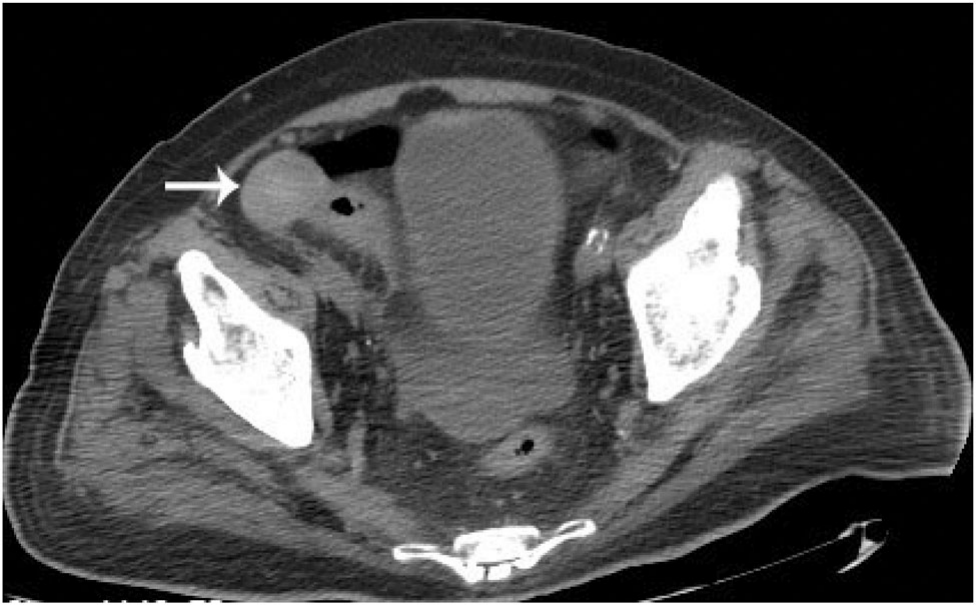
The peritoneal loose body in the abdominal CT scan after bowel perforation occurred.

**Figure 2.**
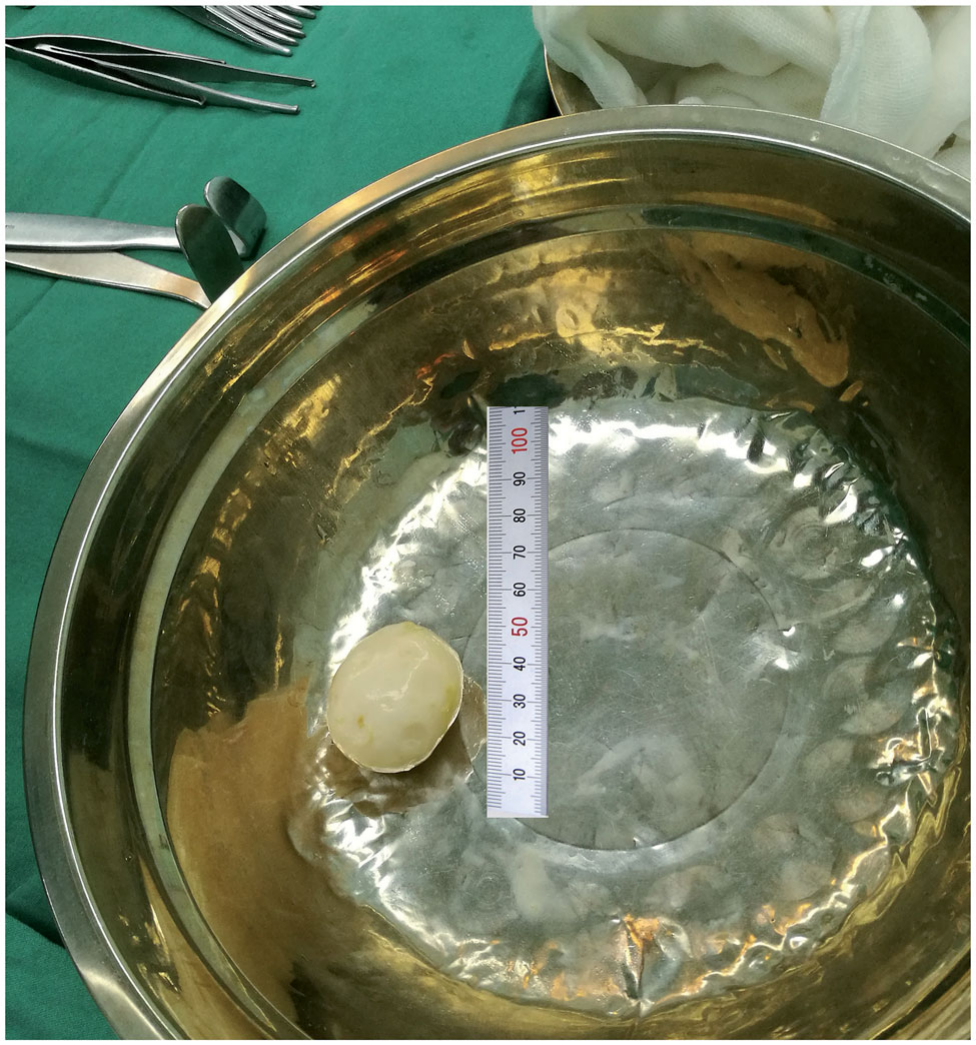
The peritoneal loose body obtained during exploratory laparotomy.

However, abdominal distension and pain occurred two days later, and the patient began to have a fever of 37.8 °C. Physical examination showed abdominal tenderness and rebound tenderness, indicating possible abdominal organ injury or peritonitis. Abdominal CT images showed pneumoperitoneum and a solid and well-defined mass around the bowel (Figure 1). In order to identify organ injuries, exploratory laparotomy was done. A penetrating wound in the sigmoid was revealed, the tip of the catheter was in the sigmoid, and, beneath the wound of the sigmoid, there was a gray and white circular mass with size of about 5 cm, with free, hard and smooth surface (Figure 2), which was diagnosed as a *peritoneal loose body* (PLB).

Sigmoidostomy was performed and the patient was moved to intensive care unit after the operation. The patient’s condition remained stable for two days after the operation; however, despite surgery and other active treatment, he succumbed on the third day because of his severe condition.

## Discussion

The PLB is rare and has not been reported previously in PD patients. The most common causes of the PLB are thought to be torsion and separation of the epiploic appendices, which are visceral peritoneal pouches full of fat that exists along with the antimesenteric tenia of the colon [3]. The PLB is usually small, varying from 0.5 to 2.5 cm in diameter; a larger PLB (larger than 4 cm in diameter) as observed in our patient is exceedingly rare [4]. Whereas a small PLB is often asymptomatic, a large PLB may present with various symptoms (abdominal pain or dyspepsia) [5,6], and it can in severe cases also cause intestinal obstruction [7] and urinary retention [8]. The initial diagnosis is difficult and a large PLB was frequently misdiagnosed preoperatively as tumorous disease [3].

In the present case, the large PLB is thought to have contributed to bowel perforation by serving as a supporting point beneath the sigmoid; the puncture needle easily went through peritoneum and the sigmoid without resistance during the catheterization, so the catheter was also guided through the sigmoid. Since it was a straight catheter (without coiled tip), the tip was dragged into the sigmoid slowly in the following 2 days, and all the symptoms and signs occurred after the penetrating wound was exposed. Besides, abdominal wall adhesion or other complicated abdominal conditions were not found during the exploratory laparotomy. Therefore, the PLB is thought to have contributed to bowel perforation in the present case.

Since it can move freely in the cavity, percutaneous PD catheter insertion should be avoided if a PLB is diagnosed before the operation, and the percutaneous operation should be replaced by open surgical dissection or surgical laparoscopy so that bowel perforation caused by the PLB will not occur.
